# Updating dopamine reward signals

**DOI:** 10.1016/j.conb.2012.11.012

**Published:** 2013-04

**Authors:** Wolfram Schultz

**Affiliations:** Department of Physiology, Development and Neuroscience, University of Cambridge, Downing Street, Cambridge CB2 3DY, UK

## Abstract

Recent work has advanced our knowledge of phasic dopamine reward prediction error signals. The error signal is bidirectional, reflects well the higher order prediction error described by temporal difference learning models, is compatible with model-free and model-based reinforcement learning, reports the subjective rather than physical reward value during temporal discounting and reflects subjective stimulus perception rather than physical stimulus aspects. Dopamine activations are primarily driven by reward, and to some extent risk, whereas punishment and salience have only limited activating effects when appropriate controls are respected. The signal is homogeneous in terms of time course but heterogeneous in many other aspects. It is essential for synaptic plasticity and a range of behavioural learning situations.

## Highlights

► Dopamine prediction errors are influenced by model-based information. ► Dopamine neurons show limited activations to punishers when proper controls are made. ► Dopamine neurons do not code salience to a substantial extent. ► Intact dopamine mechanisms are required for learning and posysynaptic plasticity.

**Current Opinion in Neurobiology** 2013, **23**:229–238

This review comes from a themed issue on **Macrocircuits**

Edited by **Steve Petersen** and **Wolf Singer**

For a complete overview see the Issue and the Editorial

Available online 22nd December 2012

0959-4388/$ – see front matter, © 2012 Elsevier Ltd. All rights reserved.

**http://dx.doi.org/10.1016/j.conb.2012.11.012**

## Introduction

The smoke signals of the American Indians and the peep-peep from Sputnik demonstrate that information processing systems use signals. The brain is no exception. Its action potentials are instrumental for extracting information from the environment and directing behaviour. The foremost function of the brain is to assure individual survival and gene propagation for ultimate evolutionary fitness. To this end individuals need to acquire specific substances for their bodily functions. The substances come in foods and liquids and require effort to obtain. They are called rewards and support learning, approach behaviour, decision making and positive emotions like pleasure, desire and happiness. Numerous other objects, events and situations have similar reward functions and contribute to evolutionary fitness. To understand reward processing, we need to study neuronal signals for these objects. We can describe reward functions in formal behavioural terms like Pavlovian and operant conditioning, habits and goal directed behaviour, economic decision making and see how alterations in brain functions influence these processes. But to understand how the brain as an information processing machine mediates reward we need to take these terms apart and identify and understand neuronal signals for their components. This review provides an update on dopamine neurons that provide a reward signal for updating economic decision variables.

## Nature of dopamine signal

Most midbrain dopamine neurons (75–80%) show rather stereotyped, phasic activations with latencies of <100 ms and durations of <200 ms following unpredicted food or liquid rewards. This response codes a quantitative prediction error, namely the difference between received and predicted reward value. A reward that is better than predicted elicits an activation (positive prediction error response), a fully predicted reward draws no response, and a reward that is worse than predicted induces a depression (negative error response). Most dopamine neurons (60–75%) respond in similar ways to reward predicting stimuli, coding positive and negative, higher order reward prediction errors at the time of the stimulus relative to the prediction at that moment [1,2,3^•^,4^••^].

## Power of negative prediction error signal

The positive prediction error response can amount to phasic tripling of impulse rate, whereas the negative response has limited dynamic range owing to the low baseline activity of dopamine neurons (3–5 impulses/s), which might suggest limited negative coding [5,6]. However, synaptic transmission is unlikely to operate on an absolute scale. Completely shutting off a signal is more noticeable than tripling it. Abruptly extinguishing illumination in a dim auditorium has more impact than increasing it threefold. The observation that the pause affects almost all dopamine neurons makes it a powerful negative signal that would phasically stop stimulation of both low affinity D1 and high affinity D2 receptors. Variations of predictions together with proper measurement of negative response intensity, as expressed by duration of depression in extracellular recordings, reveal bidirectional graded reward prediction error coding in dopamine neurons [7]. Thus the negative dopamine error signal is graded and likely to have a major impact on postsynaptic neurons.

## Temporal difference neuronal signal

**Figure 1:**
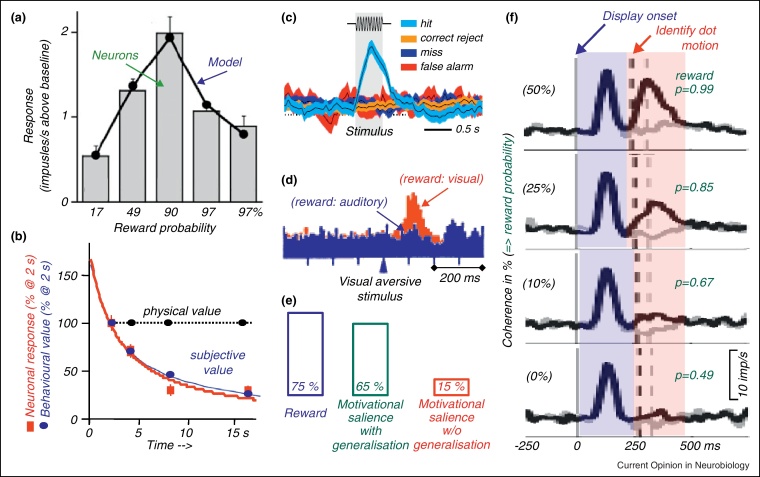
Characteristics of phasic dopamine reward prediction error responses. **(a)** Neuronal coding of reward prediction error closely parallels theoretical prediction error of temporal difference (TD) model ([4^••^], © National Academy of Sciences USA). **(b)** Temporal discounting of neuronal response to stimulus predicting differently delayed rewards closely parallels behavioural discounting ([15], © Society for Neuroscience). **(c)** Neuronal response depends on subjective stimulus perception ([24^••^], © National Academy of Sciences USA). **(d)** Stimulus generalisation explains majority of responses to conditioned aversive stimuli. Change in sensory modality of reward predicting stimulus reduces response to unchanged aversive stimulus ([34], © Nature). **(e)** Percentages of dopamine neurons activated by reward (blue, left), motivational salience uncontrolled for stimulus or context generalisation (green) and true motivational salience (red, right). Data from [34]. **(f)** Graded coding of value prediction after initial generalisation coincides with stimulus identification by animal in dot motion task. Percentage of coherently moving dots results in graded percentage of correct performance and reward delivery ([3^•^], © Society for Neuroscience).

The dopamine response to reward is compatible with the notion of primary reinforcer prediction error according to error driven learning rules [8]. These learning rules are extended by temporal difference (TD) reinforcement learning to higher order reinforcement [9]. Compatible with these notions, dopamine neurons show phasic responses to higher order prediction errors evoked by conditioned, reward predicting stimuli [1]. In a recent multistep task, monkey dopamine neurons coded the TD error quantitatively by reflecting the difference between the sum of multiple future rewards and their prediction [4^••^]. In the particular task used, the reward probabilities increase towards the end of the multistep sequence, resulting in the highest discounted sum of future reward in the centre from which the lower predictions arising from earlier stimuli are subtracted (Figure 1a). The dopamine responses match this temporal profile of TD error closely, demonstrating the most complete relationship of phasic dopamine responses to the TD model of reinforcement so far reported.

## Model-free vs. model-based reinforcement learning

Reinforcement learning establishes reward predictions that are essential for informed decision making [8]. Model-free reinforcement learning occurs through the experienced contiguity between stimuli or actions and reinforcers. However, individuals also learn about states, situations, contexts, rules, sequences, and conditional probabilities, which may be summarily called models of the world. Knowledge derived from models can be useful for improving reward predictions. The processes can include frequentist and Bayesian inferences, logical reasoning, or any method that improves the identification of relevant states. Model-based reinforcement learning involves two separate processes, the acquisition and updating of the overall model, and the influence of the model on current predictions [10,11]. Although it is currently unknown whether dopamine prediction error responses might be involved in the acquisition or updating of the models, they may incorporate the predictive information from models. As the experienced reward is physical and thus independent of the model, the model's influence on the prediction error would derive from the influence on the reward prediction.

In learning situations governed only by experienced rewards, consecutive unrewarded trials lead to progressively decreasing reward prediction. Correspondingly, positive prediction errors to an unpredicted reward increase, because the repeated absence of reward makes the reward less expected and the delivery of reward becomes more surprising. However, in particular tasks, sequences of unrewarded trials may lead to increasing reward probability (increasing hazard rate) and thus increasing reward prediction. Now the reward becomes progressively less surprising and hence induces a prediction error that decreases over successive trials. Learning in such a task benefits from knowledge about the underlying model of the world. Indeed, dopamine error responses follow precisely this scheme, decreasing with increasing numbers of trials since the last reward [12], suggesting that they process model-based predictions.

Dopamine responses are influenced by previously acquired information about reward probability distributions [13]. The error responses adapt within 2 s to the currently valid expected values and standard deviations despite ten fold differences in reward magnitude, without requiring resampling of the distributions. The adaptations probably reflect scaling of the prediction which is subtracted from the currently experienced reward value to obtain the prediction error.

Repeated reversals of stimulus-reward or position-reward associations lead to acquisition of a rule that helps individuals reverse their reactions within one trial (reversal set). Correspondingly, dopamine responses to reward predicting stimuli reverse after one trial without having experienced the contiguity between the newly valid stimulus position and the reward [14^••^]. These data suggest that dopamine neurons are influenced by predictions derived from hidden states and Bayesian inference for estimating the reversed reward probabilities.

In each of these examples, the observed dopamine prediction error responses are not fully explained by the actual experience with reward but incorporate information from models of the world. Thus the dopamine prediction error responses occur in both model-free and model-based reinforcement learning situations, without evidence so far for a role in initial model acquisition.

## Subjective value coding in temporal discounting

Individuals make choices that maximise their rewards. Although rewards have objective, physical and chemical properties, their value is defined by the needs of the individual decision maker and thus intrinsically subjective. One way to distinguish subjective from objective reward value is temporal discounting, the decrease in subjective reward value with increasing delays despite constant physical size. Psychometric analysis of choices in monkeys with variable early vs. constant 4, 8 and 16 s delayed rewards reveals graded hyperbolic decays in subjective value by 25, 50 and 75%, respectively, compared to reward at 2 s [15]. Exponential decay fit significantly less well.

Dopamine responses to reward predicting stimuli decrease monotonically across reward delays of 2–16 s in monkeys, despite the same physical amount of reward being delivered after each delay [15,16] (Figure 1b). Responses to reception of the constant reward itself show the opposite relationship, namely increasing magnitudes after increasing delays, again being fit generally better by hyperbolic than exponential functions. The increasing response profile may be owing to temporal prediction errors derived from subjective temporal uncertainty that scales with delay or magnitude prediction errors between the full size received reward and the discounted predicted value. Reward magnitude sensitive dopamine neurons in rats show higher sustained activations for several seconds following odours predicting sucrose after a short, fixed delay of 0.5 s compared to long, variable delays of 1–7 s [17].

Reward neurons in most other primary and associated reward structures of the brain show some degrees of temporal discounting, including orbitofrontal cortex [18], prefrontal cortex [19,20], dorsal and ventral striatum [21,22], premotor cortex [19] and parietal cortex [23]. Thus reward neurons code subjective rather than objective reward value in temporal discounting and thus may provide direct inputs for neuronal mechanisms underlying value based decision processes.

## Subjective stimulus coding

If dopamine neurons code reward value subjectively in temporal discounting, would they show other aspects of subjective coding compatible with the notion of an intrinsically subjective survival function of rewards? For instance, would their reward prediction coding reflect the physical presence or the subjective experience of predictive stimuli? Signal detection theory offers well proven tests. Monkeys correctly or incorrectly report the presence of stimuli (hit and false alarm, respectively), and correctly or incorrectly report stimulus absence (correct rejection and miss alarm, respectively) [24^••^]. Dopamine neurons are activated by an initial task stimulus only when the animal reports its detection, whereas they are not activated by the same physical stimulus when the animal reports its absence, nor when the stimulus is physically absent irrespective of the animal's report (Figure 1c). The data suggest that both subjective perception and physical presence of a stimulus are necessary to evoke dopamine responses, whereas physical presence alone is insufficient.

The relationship to subjective perception is also seen with dopamine error responses to subsequent cues and reward. Correct and incorrect reports of stimulus presence lead to reward in 76% of trials, whereas correct and incorrect reports of stimulus absence lead to 64% reward [24^••^]. As dopamine neurons code reward probability as value [25], we calculate the TD prediction errors at a subsequent, noninformative cue as [constant prediction by noninformative cue minus value 76] when the animal reports stimulus presence, and [constant prediction by noninformative cue minus value 64] when reporting stimulus absence. Thus the prediction error is lower when the animal reports detection compared to reporting absence of the same physical cue. Indeed, the dopamine error responses to the cue follow exactly this pattern. At reward reception, the prediction errors and the resulting dopamine responses show exactly the same difference.

Together with the responses to the near threshold stimulus itself and the subjective value coding in temporal discounting, dopamine responses reflect the animal's subjective perception and valuation of the stimuli beyond purely physical reward properties. As decisions are ultimately made according to subjective reward values, the dopamine responses may provide rather direct and parsimonious, and therefore evolutionary beneficial and selected, inputs to neuronal decision processes.

## Aversive responses

Electric shocks, painful pinches and airpuffs, as well as conditioned stimuli predicting these events, induce phasic depressions in dopamine impulse activity in anaesthetised monkeys and rodents [26–32,33^•^] and in awake monkeys, cats and rats [34–37,38^•^]. The dopaminergic nature of these neurons is verified by optogenetics [39^••^]. The depression correlates with the length of dopamine dendrites in pars reticulata of substantia nigra [40], suggesting a synaptic drive by GABAergic midbrain neurons which are excited by aversive stimuli and whose stimulation leads to depression in dopamine activity [33^•^,39^••^,41^•^].

A few dopamine neurons (5–15%) are activated by primary aversive stimuli [26,28,34], which is now confirmed [31,33^•^,36,37,38^•^,39^••^]. The overall impact of aversive stimuli on the population of dopamine neurons is difficult to assess in studies not reporting frequencies of responses relative to numbers of tested neurons or testing relatively few neurons (≤30) [32,42]. Midbrain neurons that respond to aversive events but are insensitive to the defining depressant effects of systemic apomorphine [43] are probably not dopaminergic [44,45]. Aversively activated neurons are also activated by rewards and other environmental stimuli that are not directly rewarding, and thus form a separate, limited group of highly sensitive, nonspecifically responding neurons. However, dopamine neurons that are activated by punishers do not seem to show bidirectional punisher prediction error responses [36,37]. The aversively activated dopamine neurons may constitute a distinct ventral tegmental group in rats [33^•^], although silent dopamine neurons [46] might pick up the juxtacellular staining without being activated.

Aversive stimuli increase striatal dopamine concentrations measured by *in vivo* dialysis over seconds [29,47]. The increase may reflect tonically elevated impulse activity, increased proportions of spontaneously active dopamine neurons [46] and impulse rebound after initial depressions [27,38^•^,45]. Faster voltammetry reveals striatal dopamine decreases following quinine [48] and increases in patches of striatum and nucleus accumbens core during tail pinch and accumbens shell after termination of tail pinch [49]. The increases probably reflect the limited initial and more frequent rebound dopamine activations by punishers. As termination of an aversive stimulus induces a rewarding rebound according to the opponent process theory of motivation [50], some of the dopamine increases may derive from rebound and reflect reward.

### False aversive activations

Substantial fractions of dopamine neurons (35–65%) are activated by conditioned aversive stimuli presented in random or blockwise alternation with reward predicting stimuli [34,36,37], which exceeds the frequency of activations to unpredicted primary aversive stimuli (<15%). These results are paradoxical from a reinforcement perspective because they violate a basic tenet of animal learning theory that postulates stronger motivational and attentional effects of primary, unconditioned rewards or punishers compared to stimuli conditioned to them. Although conditioned aversive stimuli may induce different forms of aversion to which dopamine neurons might be selectively sensitive, such selectivity would be surprising given their common responsiveness to all kinds of rewards. Thus other mechanisms than punishment may play a role. Closer inspections of conditioned aversive activations reveal two response components.

The initial, brief activation is identical for all conditioned reward predicting, unrewarded, differently valued and aversive stimuli, with <100 ms latency and 50–150 ms duration [2,34,51–53]. It is shorter than the full responses to reward predicting stimuli and often curtailed by subsequent depression. It leads to dopamine release in rats [54]. The responses show correlations with air puff probability across the population, which may arise from a subset of well activated neurons [37]. A proper control procedure maintains the visual aversive stimulus unchanged but changes the modality of the randomly interleaved reward predicting stimulus, for instance from visual to auditory. This manipulation reduces conditioned aversive responses from 65% to <15% of neurons [34] (Figure 1d, e), which approaches the proportion of neurons activated by primary aversive events. Thus physical similarity to rewarding stimuli may produce false aversive responses, possibly through response generalisation to rewarding stimuli. Also, reducing overall reward probability from 75% to 25% between experiments decreases the incidence of false activations by unrewarded stimuli from 63% to 1% [52,53], suggesting an effect of context conditioning (pseudoconditioning). Apparently the initial response occurs before the neuron has properly identified the stimulus.

The second response component constitutes a genuine value response [2] which differentiates well between reward and other events. It codes a graded TD reward value error, consisting of depression following aversive or unrewarded stimuli [36,51–53], and graded activations with different values [17]. The updated prediction in this response is reflected in the error response at reward time [15,52], see Figure 5 in [2].

The two response components are better separated in a dot motion task that requires time for stimulus identification [3^•^] (Figure 1f). The initial dopamine activation before stimulus identification fails to vary with value. However, it decreases when its occurrence becomes more predicted as time goes by (increasing hazard rate), thus coding a temporal prediction error. The second component is well separated and occurs 150–200 ms later when the animal identifies the motion direction that determines reward probability and thus value. It reflects the TD error between the reward value of the specific stimulus and the value predicted by the preceding fixation spot.

Taken together, the more frequent activations to conditioned aversive stimuli compared to primary punishers is probably explained by the nature of the two response components. The initial, stereotyped activation constitutes a pre-identification, higher order temporal TD error response, or possibly response generalisation to rewarding stimuli or contexts. After proper identification of the stimulus, the second component codes a TD error accurately. A fast but inaccurate initial neuronal response may provide survival advantages. It could lead to early initiation of behavioural reactions in the course of which the exact nature of the stimulus becomes identified by the second component. The mechanism would allow the agent to arrive first at the object and thus provide a slight advantage over slower competitors, which would ultimately pan out as higher evolutionary fitness. If the initial response is false and the object unrewarded, behavioural reactions can be cancelled while the second response component identifies the object 200 ms later, which would be a small price to pay.

Whereas interest in dopamine reward responses has overshadowed the long known aversive responses, some current work addresses punishment in identifiable groups of dopamine neurons without assessing their incidence relative to reward responsive neurons [32,42]. Although some dopamine neurons are definitely activated by aversive events, their proportion remains as small as 30 years ago [26,27].

## Salience

Salience refers to the capacity of stimuli to elicit arousal, alert and attention which enhance neuronal processing and behavioural reactions. There are, in principle, three forms of salience. Physical salience derives from physically strong stimuli. Novelty or surprise salience originates in novel or surprising events. Motivational salience is produced commonly by rewards and punishers. (Incentive salience refers to motivation for rewards, in contrast to learning, and thus applies to rewards. It elicits the emotion of desire, called ‘wanting’, rather than valence independent attention [55].)

Would salience explain the higher incidence of responses to conditioned aversive stimuli (35–65%) compared to unpredicted air puffs (<15%) [34,37]? The physical salience of small conditioned visual stimuli is marginal [34,36,37] and higher with air puffs and electric shocks. Thus physical salience would be higher for the unconditioned than conditioned aversive stimuli. Novelty salience should not be a factor with well trained conditioned stimuli, and surprise salience would be equally high with unpredicted conditioned stimuli and unpredicted air puffs. Motivational salience would be higher with unpredicted air puffs than with conditioned stimuli predicting air puffs. All evidence from learning experiments suggests that the highest motivational impact arises from the primary air puff. Even fully conditioned stimuli would not have higher motivational impact, which is further reduced by the delay to air puff (temporal discounting). Thus unpredicted air puff carries the highest total salience of all events, and salience would not explain the higher incidence of activations to conditioned stimuli compared to air puffs. Motivational salience would be restricted to <15% of dopamine activations, and the remaining responses should be owing to pre-identification responses described above, or possibly pseudoconditioning [56,57].

Further arguments limit motivational salience as a major factor determining dopamine responses. First, the change from visual to auditory rewarded stimuli reduces aversive dopamine activations to stimuli without reducing the salience of the unchanged aversive stimulus [34]. Second, conditioned inhibitors for reward are motivationally very salient but fail to activate most dopamine neurons when pre-identification responses are excluded [53]. Third, negative reward prediction errors are motivationally very salient but depress rather than activate dopamine neurons, including those activated by air puffs [36,37].

Whereas 70–80% of dopamine neurons code reward prediction errors, only 10–15% are activated by both rewarding and aversive events and thus motivational salience. Therefore motivational salience induces 5–8 times less dopamine activations than reward, rather than similar proportions as suggested [58]. The depressant responses to the negative value of aversive stimuli are compatible with the predominantly positive value coding nature of dopamine neurons.

## Homogenous vs. heterogeneous properties

Together with the previously described risk responses in 30% of dopamine neurons [25], dopamine neurons show several response types with unequal proportions. One large population codes reward value, and smaller populations code reward risk, motivational salience and possibly other event properties. The primarily reward related nature of phasic dopamine signals has been confirmed in the majority of optogenetically identified dopamine neurons [39^••^] and supported by the restriction of prediction error coding to reward [36,37]. Nonexclusive coding of one particular event type lies within the natural variations of biological systems whose flexibility benefits from minor deviations.

The reward responses are homogeneous in terms of latency, duration, positive monotonicity of value coding and error coding [2] and somewhat heterogeneous owing to risk and salience coding. They occur in a neuronal system with heterogeneous inputs, transmitter colocalisation, axonal projections, and receptor and reuptake transporter expression, which may lead to regionally inhomogeneous dopamine release [49,59]. The net result of optogenetic activation of midbrain dopamine neurons is rewarding, as shown by place preference conditioning and operant nose poking behaviour [60^••^,61^••^,62^•^], although regional stimulations in terminal areas might reveal fine grained heterogeneities. Despite these variations, the high percentage of dopamine neurons coding reward, and their temporal response homogeneity in all sorts of behavioural tasks, is amazing. No other brain structure has such high proportion of reward processing neurons with such low variety of task relationships, including striatum, orbitofrontal cortex and amygdala. Although complicated tasks may let dopamine responses appear complex, appropriate analysis in terms of TD error coding demonstrates their relatively simplistic and stereotyped character [4].

## Effects of dopamine signal

The bidirectional dopamine prediction error response at the time of the reward implements fully the error term of the Rescorla-Wagner learning rule [2]. The dopamine responses to higher order, reward predicting stimuli comply with the notions of efficient TD teaching signals [63]. These dopamine signals may be involved in the two main, objectively measurable reward functions. An error signal would be ideal for mediating value learning and updating for decision making. Considering the brain as a prediction machine, an error signal would also be appropriate for inducing approach behaviour and affecting decision processes.

### Postsynaptic neuronal plasticity

A viable neuronal reinforcement mechanism involves three-factor Hebbian plasticity with dopamine and glutamate synapses located at the same dendritic spines of striatal or frontal cortical neurons, the so-called synaptic triad [2,64–66]. Indeed, electrical midbrain stimulation induces dopamine D1 receptor dependent long term potentiation in striatal neurons [66]. Further evidence suggests a crucial role of dopamine in both long-term potentiation (LTP) and depression (LTD) in striatum [67–70], frontal cortex [71,72], hippocampus [73] and amygdala [74]. Iontophoretically applied dopamine puffs induce plasticity in aplysia [75].

**Figure 2:**
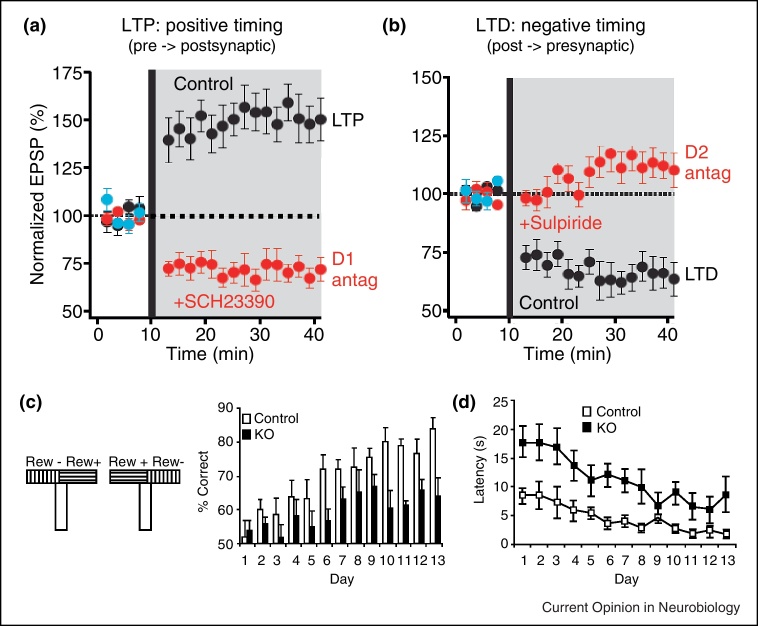
Dopamine dependency of neuronal plasticity and behavioural learning. **(a)** Positive timing in spike time dependent plasticity protocol (STDP) results in long term potentiation (LTP) at synapses from cortical inputs to striato-nigral neurons (direct pathway) (black) and is blocked by dopamine D1 receptor antagonist SCH23390 (red) ([77^••^], © Science). **(b)** Negative timing in STDP protocol results in long term depression (LTD) at cortical synapses onto striato-pallidal neurons (indirect pathway) (black) and is blocked by dopamine D2 receptor antagonist sulpiride (red) ([77^••^], © Science). **(c)** T-maze learning deficit in mice with NMDA receptor knock-out in midbrain dopamine neurons impairing dopamine burst firing ([84^•^], © National Academy of Sciences USA). **(d)** Separate performance deficit in mice tested in (c).

Protocols for spike time dependent plasticity (STDP) demonstrate LTP when presynaptic stimulation precedes postsynaptic stimulation by a few tens of milliseconds, whereas LTD occurs with reverse sequence. Intact dopamine D1 receptors are required for both forms of cortically evoked plasticity in striatal neurons involving NMDA receptors [76^••^]. When dissociating dopamine receptor localisation on striatonigral neurons (D1; ‘direct pathway’) and striatopallidal neurons (D2; ‘indirect pathway’), D1 receptors are involved in LTP in direct pathway neurons and D2 receptors are crucial for LTD in indirect pathway neurons [77^••^] (Figure 2a, b). Confirming the specificity, LTP in indirect pathway neurons does not depend on dopamine [70], which may explain dopamine independent LTP in striatal neurons not distinguished according to direct vs. indirect pathways [78]. In indirect pathway neurons, LTP is turned into LTD by additional dopamine D2 receptor stimulation [77^••^]. In hippocampus, stimulation of dopamine D1 receptors enhances STDP LTP and turns LTD into LTP [79]. Neuronal modelling demonstrates the power of a dopamine reward signal occurring a few seconds after a conditioned stimulus typical for STDP learning [80^•^].

### Presynaptic neuronal plasticity

Dopamine neurons show induction of NMDA receptor dependent LTP when a short postsynaptic burst occurs after at least 500 ms of presynaptic stimulation [81^•^]. A delay of 1 s increases LTP, whereas delays of 0 or 200 ms fail to elicit LTP, and negative delays induce LTD. The delay of 0.5–1 s may reflect the time required to achieve sufficient intracellular IP3 levels, which could constitute a molecular stimulus or eligibility trace [9] tagging synapses for modification by the postsynaptic burst. The protocol follows the requirement for behavioural conditioning in which the unconditioned stimulus (US) follows the conditioned stimulus (CS) by an optimal interval. The postsynaptic burst parallels the dopamine US response, and the NMDA receptor dependent LTP parallels the acquired dopamine CS response. Similar to behavioural and neuronal response extinction, omission of the postsynaptic burst reverses LTP back to baseline [81^•^]. Synaptic plasticity is not uniform in dopamine neurons but varies according to projection territories [42].

### Immediate effects

Dopamine exerts immediate postsynaptic effects during behavioural performance and approach behaviour. At striatal neurons of the direct pathway, dopamine has excitatory effects via the D1 receptor by eliciting or prolonging glutamate inputs and transitions to the up state (depolarisation) of the membrane potential, whereas in indirect pathway neurons D2 receptor activation has inhibitory effects by reducing glutamate release and prolonging membrane down states (hyperpolarisation) [82]. These immediate effects are synergistic with the plasticity function of dopamine in striatal LTP and LTD in direct and indirect pathway neurons [77^••^].

### Behavioural learning

Hundreds of lesioning and psychopharmacological studies using various tasks demonstrate learning deficits with impaired dopamine transmission. At the level of dopamine neurons, knock outs of burst generating NMDA receptors in mice, with resulting decreases in responses to reward predicting stimuli, lead to a range of learning deficits, including conditioned place preference, maze learning and operant conditioning, that are dissociated from performance deficits [83,84^•^,85] (Figure 2c, d). In confirmation, knock out of GABA-A receptors inhibiting dopamine neurons enhances electrically evoked dopamine release and T-maze and lever press learning [86]. At the postsynaptic level, intra-accumbens or systemic administration of dopamine D1 receptor blockers impairs simple stimulus-reward learning [87] that engages dopamine neurons, well distinguished from performance [88^••^]. Learning is also impaired by knock out of NMDA receptors on mouse striatal neurons expressing dopamine D1 receptors [89], closely following the scheme of three-factor Hebbian learning [2,64–66]. Similarly, learning but not performance deficits occur in a visual stimulus-reward association task by administration of a dopamine D1 receptor antagonist into monkey prefrontal cortex [90^•^].

By contrast, learning is less impaired by systemic D1 receptor blockade in select tasks with separate stimulus and goal locations that fail to engage dopamine neurons [88^••^] or when phasic dopamine release remains functional despite NMDA receptor knock-out [91]. Also, neurotoxic dopamine lesions leave reward devaluation learning by taste aversion intact [55]; this learning form may not engage phasic dopamine responses.

Taken together, learning depends on intact dopamine function in simple reward contiguity situations with explicit, easily identifiable rewards and conditioned stimuli that engage phasic dopamine responses. Learning may not depend on dopamine neurons if their phasic responses remain unengaged during learning. Despite this obvious conclusion, learning of tasks normally engaging dopamine responses might even proceed despite lesions when neuronal plasticity allows other learning systems to compensate within the tested time frame. Knock out of specific learning systems may lead to modified ontogenesis with even more opportunity for shifts to other systems. Given the crucial importance of reward for survival, multiple, flexible learning systems are biologically plausible and would enhance evolutionary fitness.

## References and recommended reading

Papers of particular interest, published within the period of review, have been highlighted as:

• of special interest
•• of outstanding interest
